# Exploring the Mechanism of Zhibai Dihuang Decoction in the Treatment of Ureaplasma Urealyticum-Induced Orchitis Based on Integrated Pharmacology

**DOI:** 10.3389/fphar.2021.602543

**Published:** 2021-05-10

**Authors:** Dong-hua Bin, Shi-ying Zhang, Min Zhan, Ling Li, Ying-qiu Li, Xing Zhou, Fang-guo Lu, Qing Zhou, Qing-hu He

**Affiliations:** ^1^ Surgery of traditional Chinese Medicine, The First Affiliated Hospital of Hunan University of Chinese Medicine, Changsha, China; ^2^ College of traditional Chinese Medicine, Hunan University of Chinese Medicine, Changsha, China; ^3^ Department of Traditional Chinese Medicine, Shenzhen Luohu People’s Hospital, Shenzhen, China; ^4^ Department of Traditional Chinese Medicine, The Third Affiliated Hospital of Shenzhen University, Shenzhen, China; ^5^ Medical School, Hunan University of Chinese Medicine, Changsha, China; ^6^ College of Integrated Traditional Chinese and Western Medicine, Hunan University of Chinese Medicine, Changsha, China

**Keywords:** Zhibai Dihuang decoction, ureaplasma urealyticum, orchitis, integrated pharmacological, bioinformatics, Chinese medicine, herb medicine

## Abstract

**Background:** Ureaplasma urealyticum (UU) infection is the most common cause of male infertility. Zhibai Dihuang Decoction (ZBDHD) can improve the rate of forwarding motility sperm, sperm deformity rate, seminal plasma zinc and refined berry sugar levels.

**Methods:** The potential targets of ZBDHD are obtained from The Encyclopedia of Traditional Chinese Medicine (ETCM). Orchitis-related targets were collected from the Genecards and OMIM databases. The Cytoscape and the Database for Annotation, Visualization and Integrated Discovery (DAVID) were utilized to construct and analyzed the networks. Finally, a rat model of orchitis caused by UU infection was used to detect related indicators of mitochondrial energy metabolism using TUNEL apoptosis detection technology, loss cytometry, Real-Time Quantitative Reverse Transcription PCR (qRT-PCR) and Western Blot.

**Results:** A total of 795 ZBDHD targets and 242 orchitis-related targets were obtained. The “ZBDHD- orchitis PPI network” was constructed and analyzed. ZBDHD can regulate signaling pathways and biological processes related to mitochondrial energy metabolism. The results of experimental studies have shown that ZBDHD maintains the integrity of sperm mitochondrial respiratory chain function by enhancing mitochondrial Na^+^-K^+^-ATPase and Ca2^+^-Mg^2+^-ATPase activities, promotes the synthesis of mitochondrial ATP, and improves sperm energy supply, thereby improving the motility, vitality and survival rate of sperm, and effectively improving the quality of semen in UU-infected rats (*p* < 0.05).

**Conclusion:**This study discovered the multi-pathway mechanism of ZBDHD intervention in UU-induced orchitis through integrated pharmacological strategies, which provides a reference for further research on the mechanism of ZBDHD intervention in orchitis in the direction of mitochondrial energy metabolism.

## Introduction

With the development and progress of society, the influence of many factors such as psychology, environment, diet, bad living habits, etc., human reproductive function shows a downward trend year by year, and about one-sixth of couples suffer from infertility (Moridi et al., 2020). This has an adverse effect on family harmony and social stability. Therefore, infertility has become a serious public health problem. Among the many factors that cause infertility, males account for about 30–50% (Huang et al., 2016), among which low sperm motility and reduced sperm count are important reasons for male infertility (Huang et al., 2015). Reproductive system infection is one of the important causes of male infertility, and Ureaplasma urealyticum (UU) infection is the most common (Gdoura, et al., 2007; Lee et al., 2013). The pathogenesis and treatment of low sperm motility caused by UU infection are still problems facing the medical community. Due to the continuous increase of antibiotic resistance and the existence of the blood-testis barrier, the efficacy of antibiotics in the treatment of UU infectious infertility has been affected. Although it has a certain effect in killing UU, it is not effective in improving sperm motility (Zhu et al., 2012; Kokkayil and Dhawan, 2015; He et al., 2016). Therefore, an important topic in the field of male diseases is to reveal its pathogenesis in depth and to find efficient, stable and durable treatments (Wang, 2017). Traditional Chinese medicine (TCM) treatment is unique in that it emphasizes a holistic view, uses multiple channels for adjustment, and two-way balance adjustment. It is of great significance to use TCM to prevent and treat UU infectious infertility to enhance sperm “vitality” (Bin et al., 2016; Wang, 2017).

Zhibai Dihuang Decoction (ZBDHD) comes from “The Golden Mirror of Medicine”. Current research shows that: ZBDHD is a classic prescription for nourishing yin and nourishing kidneys, clearing heat and reducing fire, and it has significant clinical treatment effects on various diseases (such as urinary system, endocrine system, gynecology, andrology, pediatrics, skin diseases, and venereal diseases, etc.) (Gao et al., 2020; Wu et al., 2020). The pharmacological mechanisms involved in ZBDHD include lowering blood sugar, enhancing immunity, anti-oxidation, anti-fatigue, regulating neuroendocrine, anti-tumor, etc. (Liu et al., 2018a; Liu et al., 2018b; Gao et al., 2020; Wu et al., 2020). Our previous research also found that ZBDHD can improve the rate of forwarding motility sperm, sperm deformity rate, seminal plasma zinc and refined berry sugar levels (Sheng and He, 2019; Li et al., 2019). However, its specific mechanism is still unknown.

**FIGURE 1:**
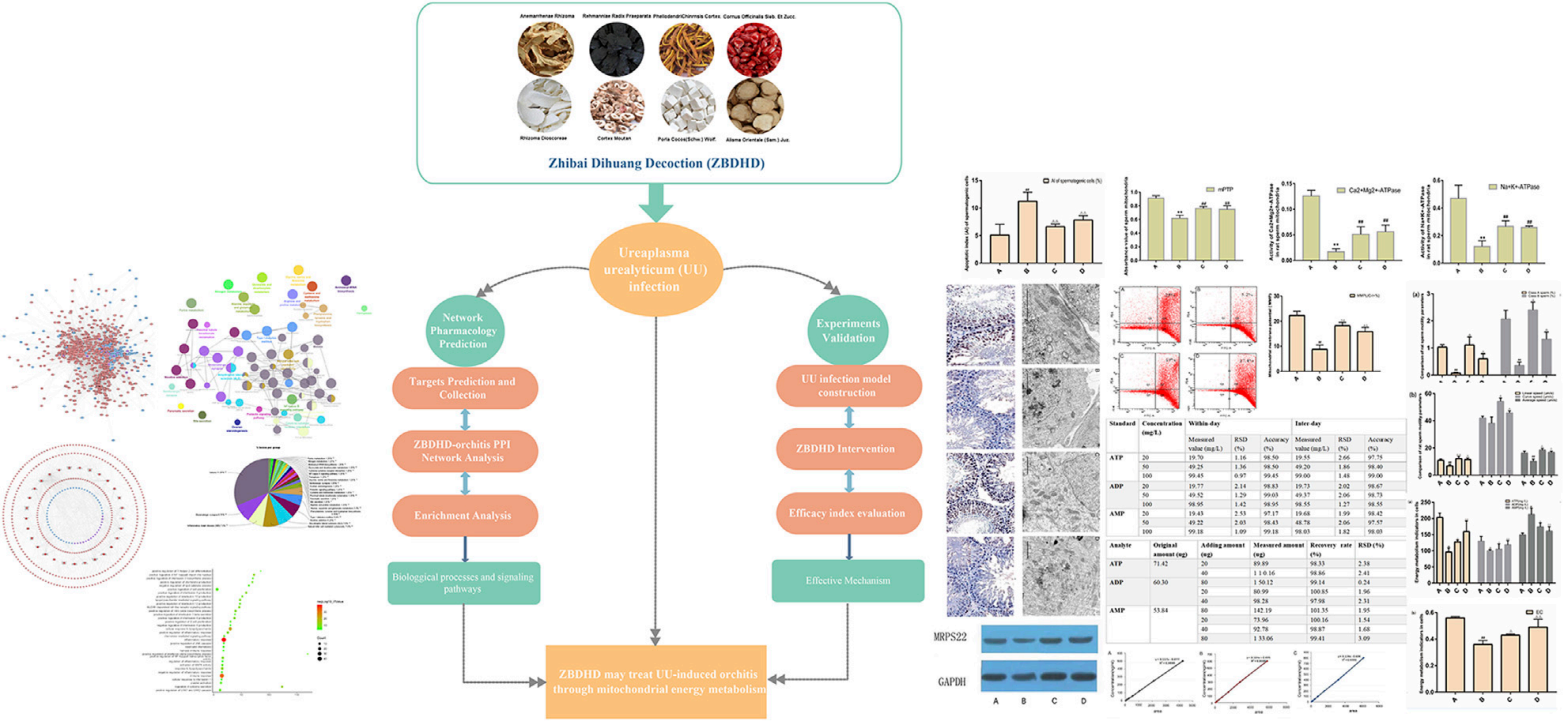
Flow chart of this research.

At present, integrated pharmacology, as a new discipline, is a discipline that studies the interaction of multi-component drugs with the body and its integration laws and principles of action (Zeng and Yang, 2017). It emphasizes multi-level and multi-link integrated research such as “whole and part,” “*in vivo* and *in vitro*,” “*in vivo* process and activity evaluation,” which is in line with the overall and systematic treatment of TCM (Yang et al., 2020; Zeng et al., 2020). Previously, we have explored the mechanism of multi-component Chinese herbal medicines in infectious diseases, endocrine diseases and immune diseases by using integrated pharmacology (Zhang et al., 2020a; Zhang et al., 2020b). In this study, we would use the strategy of integrating pharmacology and multi-directional pharmacology to study the mechanism of ZBDHD intervention in UU-induced orchitis. The process of this research is shown in Figure 1.

## Materials and Methods

### Potential Targets and Orchitis-Related Targets Collection

The potential targets of ZBDHD were collected from the Encyclopedia of Traditional Chinese Medicine (ETCM, http://www.tcmip.cn/ETCM/index.php) (Xu et al., 2018). ETCM is a database that include comprehensive and standardized information for the commonly used herbs and formulas of TCM, as well as their ingredients. The Orchitis-related targets were collected from Genecards (http://www.genecards.org) (Stelzer et al., 2018), andOnline Mendelian Inheritance in Man (OMIM) (http://omim.org/) databases (Hamosh et al., 2015). The proteins were introduced into UniProt (https://www.uniprot.org/) to correct their official gene symbols. Finally, a total of 795 ZBDHD targets and 242 orchitis-related targets were obtained (Supplementary Tables S1, S2).

### Network Construct and Analysis Methods

The protein-protein interaction (PPI) information of ZBDHD targets and orchitis-related targets were collected from String 11.0 (https://string-db.org) Szklarczyk et al., 2015). The drug target-disease target PPI network (such as ZBDHD-orchitis PPI network) were constructed by Cytoscape 3.7.2 (www.cytoscape.org/) (Bader and Hogue, 2003). The ZBDHD-orchitis PPI network was analyzed by the “Network Analyzer” and “MCODE” plug in of Cytoscape to obtain the degree of each node and clusters of this network. Finally, the Database for Annotation, Visualization and Integrated Discovery (DAVID) ver 6.8 (https://david.ncifcrf.gov/) and the “ClueGO”, plug-in of Cytoscape, were utilized to perform Kyoto Encyclopedia of Genes and Genomes (KEGG) pathway enrichment analysis and Gene Ontology (GO) enrichment analysis (Huang et al., 2009).

### Experimental Materials

#### Instruments and Reagents

The UU standard strain and UU culture medium were provided by the Department of Microbiology, Nanhua University, and the reagents required for electron microscopy were provided by the Electron Microscopy Room of Xiangya Medical College, Central South University. Mitochondrial membrane potential (mitochondrial membrane potential, MMP) detection reagent JC-1, PCR kit, and reverse transcription kit were all purchased from Kilton Biotechnology (Shanghai) Co., Ltd. BCA protein quantification kit, mouse anti-rat MRPS22 monoclonal antibody, and HRP-labeled goat anti-mouse IgG were purchased from Biyuntian Biotechnology Co., Ltd. C 18 column Diamonsil, sum, 250 × 4.6 mm; ADP reference substance (SIGMA company A2754, content 95%), ATP reference substance (SIGMA company A26209, the content is 99%), AMP reference substance (SIGMA company 01930, content is 99%). CytC ELISA kit (BOSTER Biological Technology co. ltd.). Loganin reference substance (China Institute for the Control of Pharmaceutical and Biological Products, batch number 111640-201602). Tissue Mitochondrial Isolation Kit (Cat. No.: 050217171026, Biyuntian Biotechnology Company). Purified mPTP Colorimetric Detection Kit (Cat. No.: 6-4267-11, GENMED Technology Company).

Azithromycin tablets were purchased from CSPC Ouyi Pharmaceutical Company (Lot No.: 001120941), and were formulated with normal saline to make 25 mg/ml. ZBDHD is composed of *Rehmanniae Radix Praeparata, Cornus Officinalis Sieb. Et Zucc., Rhizoma Dioscoreae, Alisma Orientale (Sam.) Juz., Cortex Moutan, Poria Cocos(Schw.) Wolf., Anemarrhenae Rhizoma, Phellodendri Chinrnsis Cortex* with ratio 24:12:12:9:9:9:6:6. The medicinal materials are provided by the First Affiliated Hospital of Hunan University of Chinese Medicine. They are appraised by Chinese pharmacists and processed in strict accordance with traditional methods. The medicinal materials are decocted in distilled water. Finally, ZBDHD was concentrated to 1 g crude drug/ml, and stored in a refrigerator at 4°C for later use.

HT7700 transmission electron microscope (Hitachi Inc.), FACSAria flow cytometer (BD Inc.), electrophoresis (Bio-Rad), ABI-7300 Real-time detector (ABI Inc.). Other instruments are provided by the Central Laboratory of Hunan University of Chinese Medicine. High performance liquid chromatograph (HPLC) (Waters company, model Waters 1525); UV detector (U.S. Waters company, model Waters 2489).

#### Experimental Animals

60 specific pathogen free (SPF) SD male rats were purchased from Hunan Slack Jingda Experimental Animal Company, 4–5 months old, weight (208 ± 15) g, laboratory animal production license number: SCXK (Xiang) 2011-0003. The animals were kept in the Special Animal Room for Pathogenic Microorganisms of Hunan University of Chinese Medicine [Permit No.: SYXK (Xiang) 2009-0001]. All animal care and use procedures comply with the National Institutes of Health (NIH) guidelines for the care and use of experimental animals and have been approved by the Institutional Animal Ethics Committee of Hunan University of Chinese Medicine.

### ZBDHD Quality Control by HPLC

#### Preparation of Sample Solution

Loganin solution: The loganin reference substance was added to 50% methanol to make a reference solution containing 50 μg per 1 ml.

ZBDHD solution: ZBDHD solution was prepared according to the aforementioned method.

#### HPLC Condition

**FIGURE 2:**
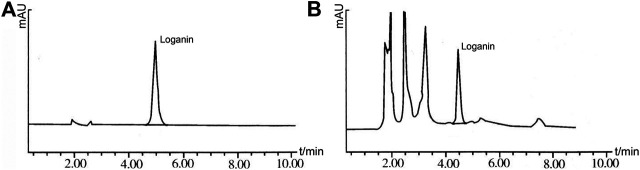
The results of HPLC. **(A)**: Loganin solution; **(B)**: ZBDHD solution.

Shim-Pack CLCODS column (150 mm × 4.6 mm, 5 μm); acetonitrile: water (15:85) as mobile phase, flow rate 1.0 ml·min-1, detection wavelength 232 nm, column temperature at room temperature. Under these conditions, the loganin peak in the sample chromatogram reached baseline separation without interference (Figure 2).

### Experimental Methods

#### Animal Modeling

First, UU titer titration was performed. The UU standard strain (freeze-dried product) was resuscitated, inoculated into liquid culture medium under aseptic conditions, cultured at 37°C for 16–24 h, and the logarithmic phase bacteria were taken. When the culture medium appears orange-red and clear bacterial liquid, the multiple dilution was implemented. The color change unit of the bacterial liquid with a titer of 10*10^6^ ccu/ml is used in the *in vitro* experiment. Then, the UU standard strain suspension was injected into the bladder to establish a UU infection animal model. The rats were fasted for 12 h before modeling. After the rats were anesthetized with 2% pentobarbital sodium (0.2 ml/100 g) and disinfected, the abdominal cavity was opened and the bladder was freed. The residual urine in the bladder was drained with a syringe, and 2 ml of normal saline or UU standard strain was injected into the bladder, and finally the abdominal cavity was sutured.

After the inoculation, the testicular puncture fluid was taken after routine feeding for 7 days and inoculated in UU culture medium at 37°C and 5% CO2. If the culture medium turns from yellow to red and is transparent and clear, 0.2 ml UU is transferred to solid medium after filtering through a microporous membrane. After culturing at 37°C and 5% CO2 for 72 h, the colonies were observed under a low-power microscope to confirm whether there was UU growth in the testicular tissue.

#### Animal Grouping and Intervention

Successfully modeled UU-infected rats were randomly divided into model group, ZBDHD group, and positive control group. At the same time, normal rats were used as normal group, with 15 rats in each group. Gavage was started on the 10th day after the successful model building, once daily. According to Sellami et al., 2014, (Guo et al., 2017) the dose of ZBDHD group is 1 g/(kg d) [i.e. ZBDHD 1 ml/(kgd)], the dose of positive control group is 0.105 g/(kg d) [i.e. azithromycin suspension 1 ml/(kgd)]. The normal group and the model group were given corresponding volume of normal saline by gavage. The testes of the rats were removed, the testis capsule and blood vessels were thoroughly stripped, and the testes were placed in a cell culture dish, and 3 ml PBS was added to completely soak the testes for later use.

#### Detection of Spermatogenic Cell Apoptosis

The prepared rat testis tissue sections were tested for spermatogenic cell apoptosis according to the instructions of the TUNEL detection kit. The cells with brown particles in the nucleus were apoptotic cells. Twenty seminiferous tubules were randomly selected from each section, and the number of positive spermatogenic cells apoptosis in the total spermatogenic cells was counted, and the positive cell rate was calculated, which was the apoptotic index (AI).

#### Preparation of Spermatogenic Cell Suspension and Sperm Motility Test

The testicular tissue was collected under aseptic conditions to prepare a spermatogenic cell suspension, which was placed in a 32°C, 5% CO2 incubator for 5 min to diffuse for use. Sperm suspension was diluted with PBS solution in a ratio of 1:9, fully shaken. Color sperm dynamic detection system was used to measure sperm motility parameters.

#### Detection of MMP and Mitochondrial Structure of Spermatogenic Cells

Alexa Fluor 488 and R-phycoerythrin are excited by the 488 nm light of the flow cytometer to detect the fluorescence signal at 520 nm and analyze the MMP of spermatogenic cells. A long strip specimen of the middle part of the left testis of the rat was taken out, and ultra-thin sections were made according to the requirements of electron microscopy. The ultra-thin sections were stained with uranyl acetate and lead nitrate, and then electron microscopy observations and photographs were performed to observe the mitochondrial structure of spermatogenic cells.

#### Detection of MRPS22 mRNA in Spermatogenic Cells by Real-Time Quantitative Reverse Transcription PCR

The total RNA of the spermatogenic cell suspension was extracted and subjected to RTPCR detection. The data obtained in the experiment was analyzed by ABI’s own RQ software, and the relative quantification of target gene expression was analyzed by the 2-ΔΔCt method. Each sample was repeated 3 times in parallel, and the average was taken for analysis.

#### Detection of MRPS22 Protein in Spermatogenic Cells by Western Blot

The spermatogenic cell suspension was lysed and homogenized, and the MRPS22 protein of spermatogenic cells was detected by Western blotting. Using *β*-actin as the internal reference, the Image-pro Plus processing software was used to analyze the image information and calculate the relative expression level.

#### Quantitative Detection of ATP, ADP, AMP in Sperm Mitochondria

A random number table was used to select 5 rats in each group, and the contents of ATP, ADP, and AMP in sperm mitochondria were detected by HPLC, and proceeded in accordance with the procedures of each kit. Energy charge (EC) value: EC = (ATP +0.5 ADP)/(ATP + ADP + AMP).

#### Determination of Sperm CytC Content by ELISA

The content of CytC in rat testis tissue was tested according to the operation steps of the ELISA test kit.

#### Mitochondrial Permeablity Transition Pore Opening Detection

After the purified mitochondrial sample to be tested was prepared, 100 μl of the mitochondrial sample (a total of 0.2 mg) was transferred to a 1.5 ml centrifuge tube, 10 μl of staining solution (Reagent A) was added, and the mixture was evenly mixed. Then, it was incubated in a cell incubator at 37°C in the dark for 15 min, and then centrifuged in a miniature benchtop centrifuge at 4°C for 5 min at a speed of 16,000 g, and the supernatant was removed. Then the pre-heated preservation solution (Reagent C) at 37°C was added to mix the particles. Finally, 100 μl of the above suspension was transferred to a black 96-well plate, and put into a fluorescence microplate reader for detection (Excitation wavelength 488 nm, emission wavelength 505 nm). If the relative fluorescence units (RFU) decreases, it indicates that mPTP is enhanced.

#### Sperm Mitochondrial Na^+^-K^+^-ATPase, Ca^2+^-Mg^2+^-ATPase Detection

After the sperm suspension is prepared, the sperm mitochondria are separated by a differential fractionation method and placed in an ice tank for later use. The determination of Na^+^-K^+^-ATPase and Ca^2+^-Mg^2+^-ATPase activity is strictly in accordance with the instructions of the mitochondrial Na^+^-K^+^-ATPase and Ca^2+^-Mg^2+^-ATPase kit instructions.

### Statistical Analysis

SPSS 17. 0 is used for statistical analysis, and the measurement data is expressed as x ± s. The independent sample t test was used to compare the difference in means between two groups. One-way ANOVA is used to compare the mean difference between multiple groups, and the LSD test is used for pairwise comparison between multiple groups. Pearson analysis was used to analyze the correlation between MRPS22 and MMP. All tests are two-sided tests, and *p* ≤ 0.05 is considered statistically significant.

## Results

### ZBDHD-Orchitis PPI Network Analysis

#### ZBDHD-Orchitis PPI Network Construction

**FIGURE 3:**
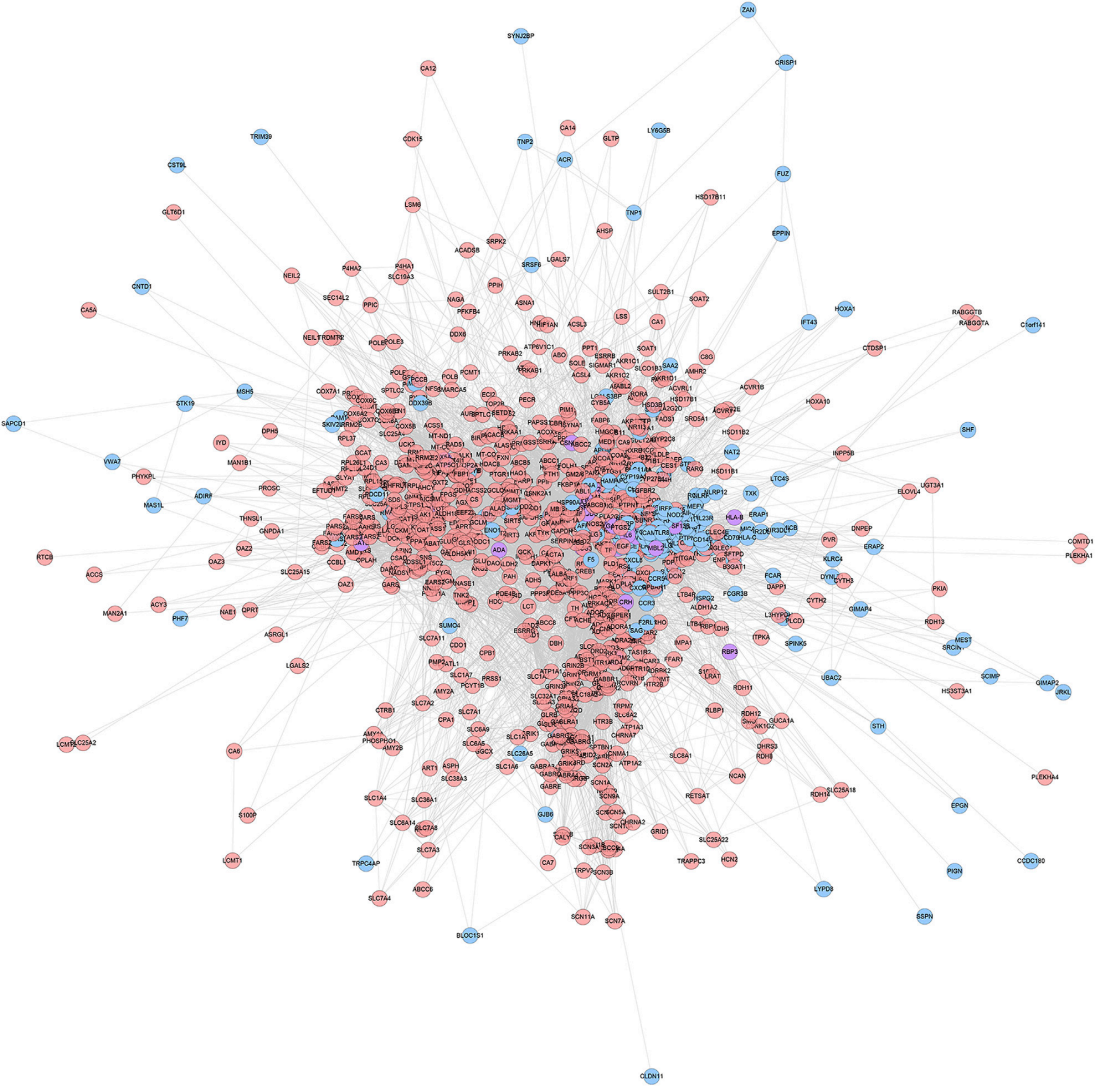
ZBDHD-orchitis PPI Network (Pink circles stand for ZBDHD target, Blue circles stand for orchitis targets, purple circles stand for ZBDHD-orchitis target).

ZBDHD-orchitis PPI network consists of 946 nodes (726 ZBDHD target nodes, 194 orchitis target nodes and 26 ZBDHD-orchitis target nodes) and 18,098 edges (Figure 3). The targets are arranged in descending order according to their degree, the top 20 can be divided into 3 category: 1) ZBDHD target: GAPDH (294 edges), INS (290 edges), AKT1 (253 edges), MAPK3 (199 edges), SRC (166 edges), EGF (157 edges), CASP3 (153 edges); 2) Orchitis related targets: ALB (276 edges), CXCL8 (188 edges), IL10 (180 edges), STAT3 (167 edges), CAT (160 edges), IL2 (158 edges), IL4 (151 edges), HSP90AA1 (147 edges); 3) ZBDHD-orchitis target: IL6 (249 edges), TNF (227 edges), VEGFA (192 edges), IL1B (173 edges), TLR4 (168 edges).

#### Biological Processes of ZBDHD-Orchitis PPI Network

**FIGURE 4:**
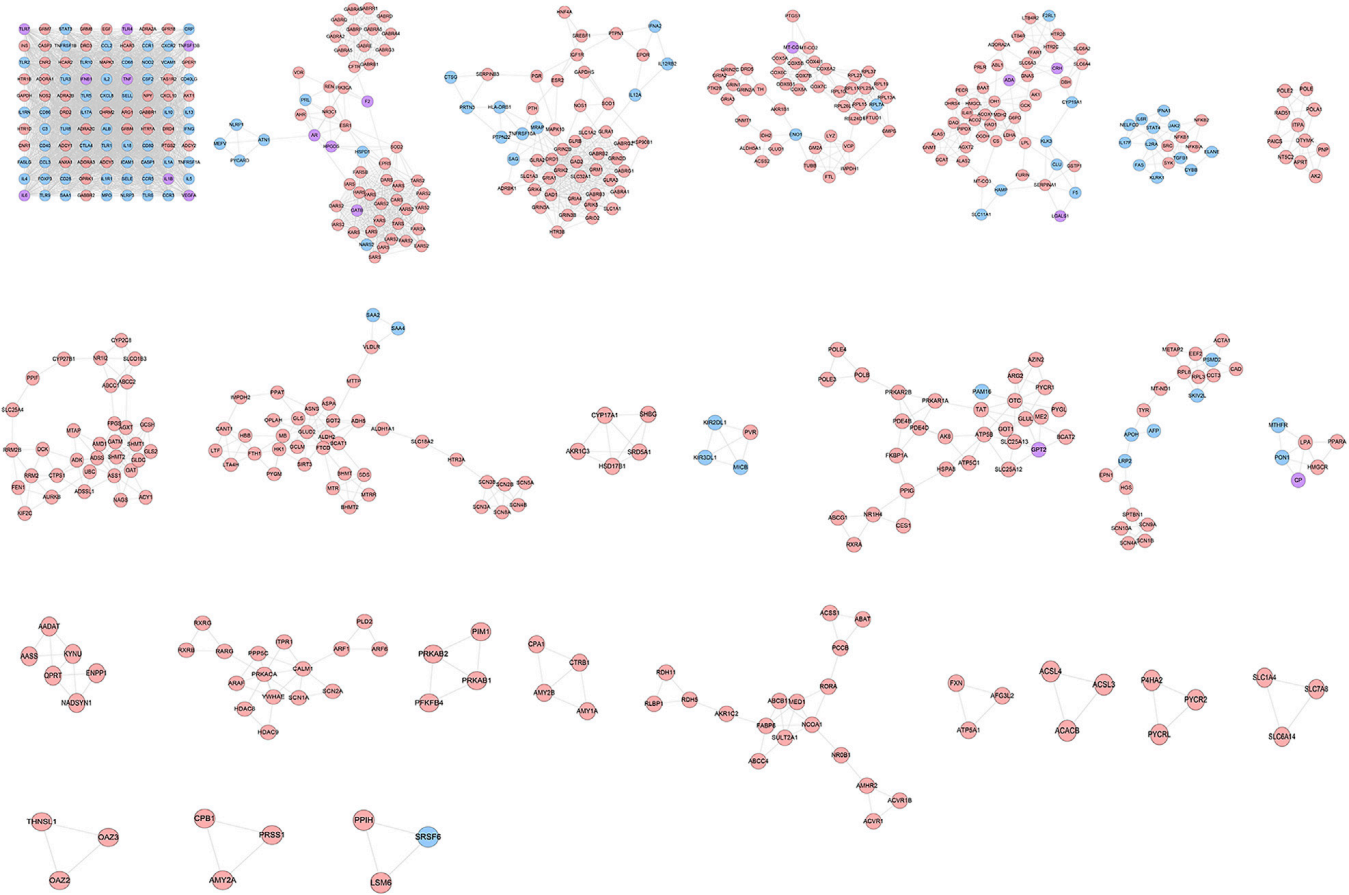
Clusters of ZBDHD-orchitis PPI Network (Pink circles stand for ZBDHD target, Blue circles stand for orchitis targets, purple circles stand for ZBDHD-orchitis target).

The ZBDHD-orchitis PPI network was analyzed by MCODE and 27 clusters were obtained (Figure 4 and Table 1). The targets and genes of top 10 clusters were input into DAVID and ClueGO to perform GO enrichment analysis so as to obtain the biological processes of each cluster.

**TABLE 1:** Clusters of DGBXD known Target-AS PPI Network.

Cluster	Score	Nodes	Edges	Targets and genes
1	56.878	99	2,787	IL18, ADORA1, IL1RN, GAPDH, ADORA3, CD40LG, IL4, CXCL8, NOS2, TLR2, CASP1, MPO, NLRP3, FOXP3, IL2, IL17A, CD68, IL1A, IL1R1, IL13, SAA1, IFNG, ALB, ICAM1, FASLG, TNFRSF1B, INS, VCAM1, IL5, ARG1, MAPK3, SELL, TLR4, TLR7, IL1B, TLR3, CCL2, CD40, CXCL10, CXCR2, C3, CCR3, CD28, CNR1, CNR2, TLR1, TNF, TLR8, TLR5, CCR5, CTLA4, TLR9, NPY, GPR18, CSF2, HTR1A, HTR1B, SELE, HTR1D, ADRA2A, ADRA2B, TLR6, ADRA2C, CD86, GPER1, CD80, TLR10, GRM4, CCL3, GRM7, GRM8, CHRM2, IL6, IFNB1, PTGS2, GABBR1, GABBR2, ANXA1, CASP3, TNFSF13B, AKT1, VEGFA, TAS1R2, STAT3, EGF, ADCY1, ADCY2, ADCY5, DRD2, DRD3, DRD4, OPRK1, IL10, TNFRSF1A, NOD2, HCAR2, HCAR3, CRP, CCR1
2	17.309	56	476	GABRP, GABRQ, AR, PIK3CA, LARS, GABRR1, TARS, GABRB1, REN, CARS2, AHR, F2, GARS, CARS, GATB, NLRP1, YARS, ATN1, PYCARD, FARS2, EARS2, ESR1, HARS, DARS, VARS, PARS2, YARS2, PRL, FARSA, FARSB, TARS2, IARS2, IARS, AARS, LARS2, GABRG3, AARS2, VDR, HSPD1, DARS2, SOD2, GABRA2, GABRA3, GABRA4, GABRA5, GABRA6, NR3C1, KARS, SARS, NARS2, MEFV, HPGDS, EPRS, CFTR, GABRD, GABRE
3	12.231	53	318	HNF4A, NOS1, SLC32A1, IGF1R, PTPN1, GABRB2, GABRB3, IFNA2, GAD1, GAD2, GRID2, PGR, PRTN3, ESR2, EPOR, MRAP, GRIN3A, GRIN3B, HSP90B1, GABRG1, GABRG2, PTH, SLC1A1, SLC1A2, SLC1A3, GRIK2, HLA-DRB1, GRIK4, SOD1, GRIK5, PTPN22, GLRB, GRIA1, ADRBK1, GLRA1, GLRA2, GLRA3, TNFRSF10A, GRIA4, GAPDHS, HTR3B, DRD1, GABRA1, SERPINB3, CTSG, MAPK10, SAG, IL12RB2, GRIN2B, GRIN2D, SREBF1, GRM1, IL12A
4	8.311	46	187	COX5A, ENO1, VCP, COX5B, FTL, GM2A, MT-CO1, GRIN1, MT-CO2, GRIA2, GRIA3, COX8A, PTGS1, COX6C, PTK2B, COX6B1, LYZ, COX4I1, RPL11, RPL23A, GMPS, RPL15, TUBB, COX6A2, DNMT1, EFTUD1, DRD5, GRIN2A, ACSS2, RPL19, RPL37, TH, COX7B, IDH2, GRIN2C, COX7C, RPL13A, RPL26L1, RPL7A, AKR1B1, GLUD1, RPL23, IMPDH1, ALDH5A1, RSL24D1, RPL10L
5	6.72	51	168	GSTP1, ACO2, LTB4R2, SLC6A2, SLC6A3, SLC6A4, MT-CO3, FURIN, HTR2B, DHRS4, HTR2C, GNMT, ADA, GNAS, IL4I1, SERPINA1, AGXT2, DAO, CRH, F2RL1, PIPOX, LGALS1, ADORA2A, CYP19A1, CLU, KLK3, LDHA, PRLR, PECR, LTB4R, AK1, SLC11A1, ALAS1, ALAS2, F5, GCK, FFAR1, DBH, BAAT, ACOX1, GCAT, HAO1, G6PD, OGDH, HAMP, LPL, ABL1, CS, IDH1, MDH2, HMGCL
6	6	17	48	SYK, NFKBIA, SRC, IFNA1, NFKB1, IL6R, CYBB, NFKB2, ELANE, TGFB1, NELFCD, JAK2, IL17F, FAS, IL2RA, KLRK1, STAT4
7	6	11	30	POLE2, POLA1, DTYMK, PAICS, PNP, NT5C2, APRT, AK2, RAD51, POLE, ITPA
8	5.438	33	87	GLDC, FPGS, KIF2C, UBC, SHMT1, FEN1, SHMT2, GLS2, ADSSL1, ABCC1, ASS1, ABCC2, ADK, CTPS1, SLCO1B3, ACY1, CYP2C8, NAGS, RRM2B, AURKB, DCK, NR1I2, CYP27B1, GATM, MTAP, OAT, SLC25A4, GCSH, AMD1, PPIF, RRM2, AGXT, ADSS
9	4.833	25	58	IL23R, TNFAIP3, ITGB2, HSP90AA1, PLG, NOS3, CD4, IRF8, LCK, B2M, ITGAL, CAMP, CREB1, PPARG, S100B, IL12RB1, LCN2, THBD, GPT, LGALS3, CD1E, KITLG, CAT, ACE, CTSB
10	4.718	40	92	ALDH1A1, SCN5A, SIRT3, ASNS, OPLAH, BHMT, GOT2, PPAT, FTCD, ALDH2, HBB, MTR, SCN3A, SCN3B, LTF, SLC18A2, HTR3A, SDS, CANT1, GCLM, ASPA, GLS, SAA4, BCAT1, MTRR, PYGM, SAA2, ADH5, LTA4H, VLDLR, GLUD2, BHMT2, FTH1, IMPDH2, MTTP, SCN8A, SCN2B, MB, HK1, SCN4B
11	4.5	5	9	SHBG, SRD5A1, AKR1C3, HSD17B1, CYP17A1
12	4	4	6	PVR, KIR2DL1, MICB, KIR3DL1
13	3.933	31	59	PRKAR2B, GOT1, ATP5C1, RXRA, HSPA8, OTC, CES1, TAT, AK8, SLC25A12, PAM16, SLC25A13, ATP5B, ABCG1, GLUL, PDE4B, PDE4D, PYCR1, NR1H4, FKBP1A, POLB, ARG2, PYGL, BCAT2, POLE3, POLE4, PPIG, GPT2, ME2, AZIN2, PRKAR1A
14	3.9	21	39	ACTA1, SCN1B, MT-ND1, TYR, SKIV2L, HGS, PSMD2, CCT3, LRP2, CAD, SCN10A, EEF2, AFP, RPL3, METAP2, SPTBN1, RPL8, APOH, SCN4A, SCN9A, EPN1
15	3.6	6	9	PPARA, LPA, CP, HMGCR, PON1, MTHFR
16	3.6	6	9	AADAT, KYNU, AASS, NADSYN1, ENPP1, QPRT
17	3.333	16	25	YWHAE, ITPR1, SCN1A, CALM1, PLD2, RARG, HDAC8, HDAC9, RXRB, RXRG, ARAF, PRKACA, PPP5C, ARF1, SCN2A, ARF6
18	3.333	4	5	PRKAB1, PRKAB2, PFKFB4, PIM1
19	3.333	4	5	CPA1, AMY1A, AMY2B, CTRB1
20	3.059	18	26	AKR1C2, ACVR1B, ACVR1, NCOA1, RDH5, ABAT, MED1, PCCB, ACSS1, FABP6, RDH11, NR0B1, RLBP1, ABCB11, AMHR2, ABCC4, RORA, SULT2A1
21	3	3	3	ATP5A1, FXN, AFG3L2
22	3	3	3	ACSL4, ACACB, ACSL3
23	3	3	3	PYCR2, PYCRL, P4HA2
24	3	3	3	SLC6A14, SLC7A8, SLC1A4
25	3	3	3	OAZ3, THNSL1, OAZ2
26	3	3	3	CPB1, PRSS1, AMY2A
27	3	3	3	PPIH, SRSF6, LSM6

**FIGURE 5:**
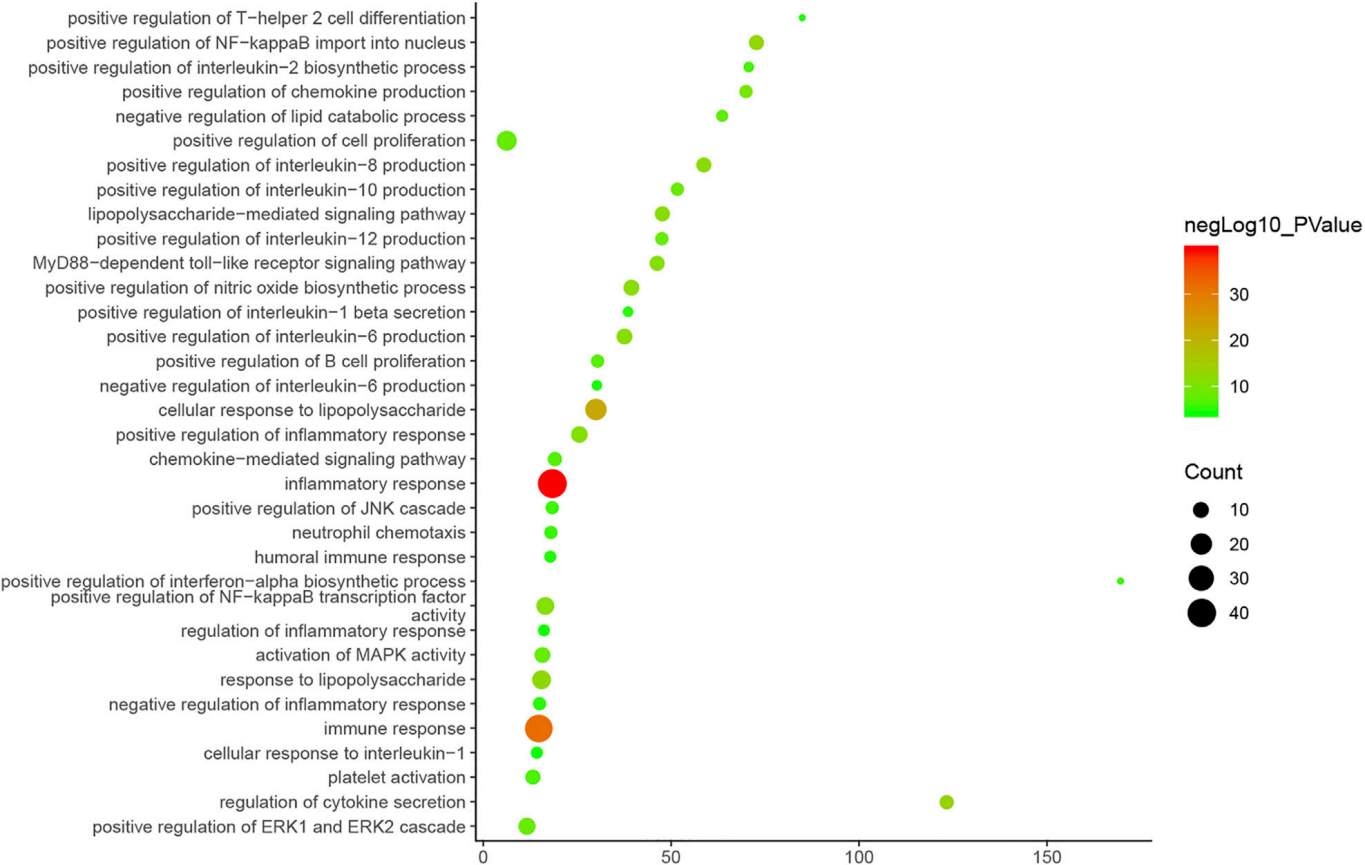
Bubble chart of biological processes in cluster 1 (X-axis stand for fold enrichment).

Cluster 1, 6 and 9 is related to inflammation and immune response. Cluster 2, 4 and 5 are associated with mitochondria and energy metabolism. Cluster 8 are associated with mitochondria and nutrient metabolism. Cluster 3 and 10 did not return any orchitis-related biological processes (Supplementary Table S3). The *p*-value, fold enrichment and count of biological processes in cluster 1 were shown in Figure 5 as an example.

#### Pathway of ZBDHD-Orchitis PPI Network

**FIGURE 6:**
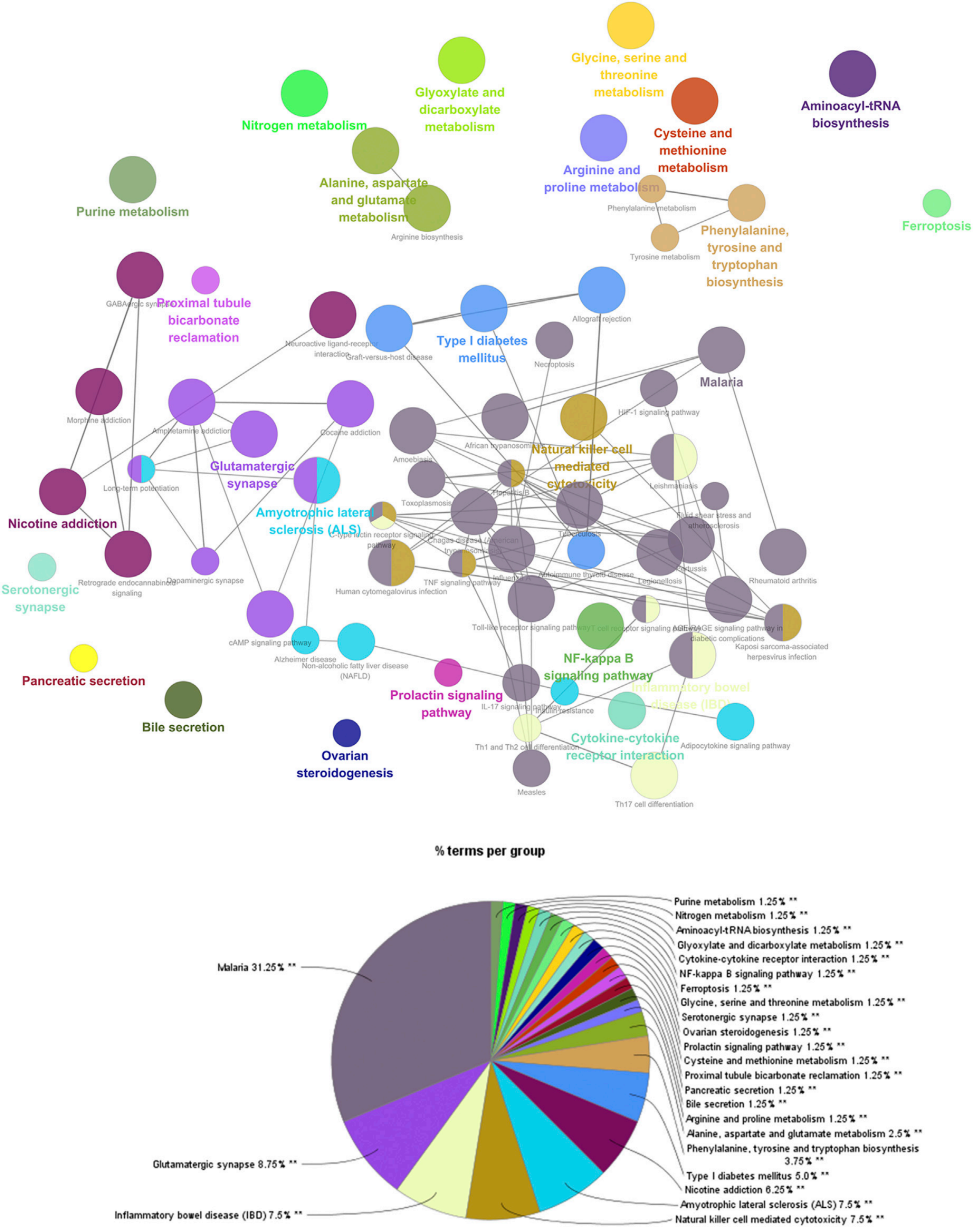
The results of ClueGO.

The targets in ZBDHD- orchitis PPI network were input into DAVID and ClueGo to perform pathway enrichment analysis and a lot of human signaling pathways returned (Figure 6).

**FIGURE 7:**
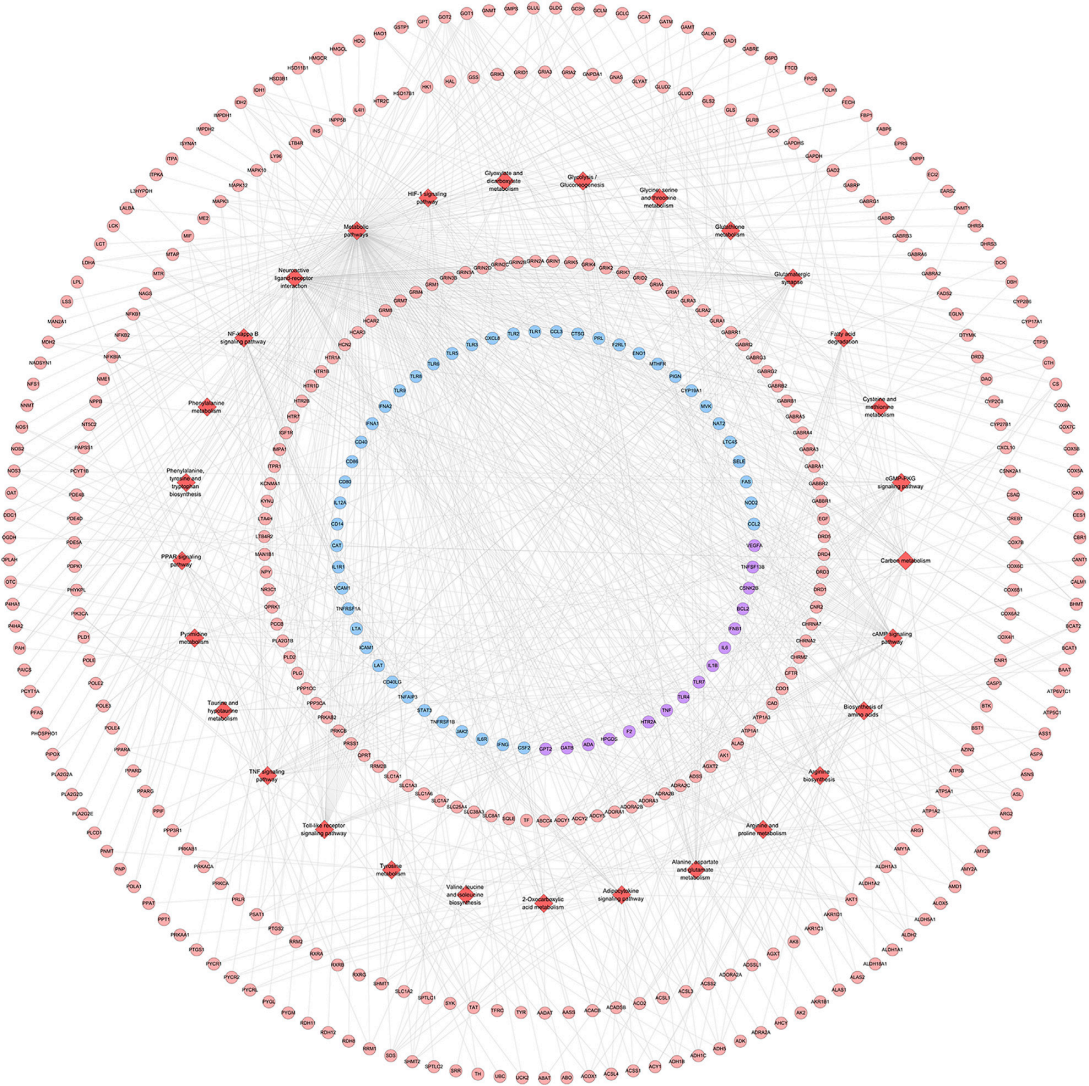
ZBDHD-orchitis PPI Network (Red diamond stand for signaling pathway. Pink circles stand for ZBDHD target, Blue circles stand for orchitis targets, purple circles stand for ZBDHD-orchitis target).

**FIGURE 8:**
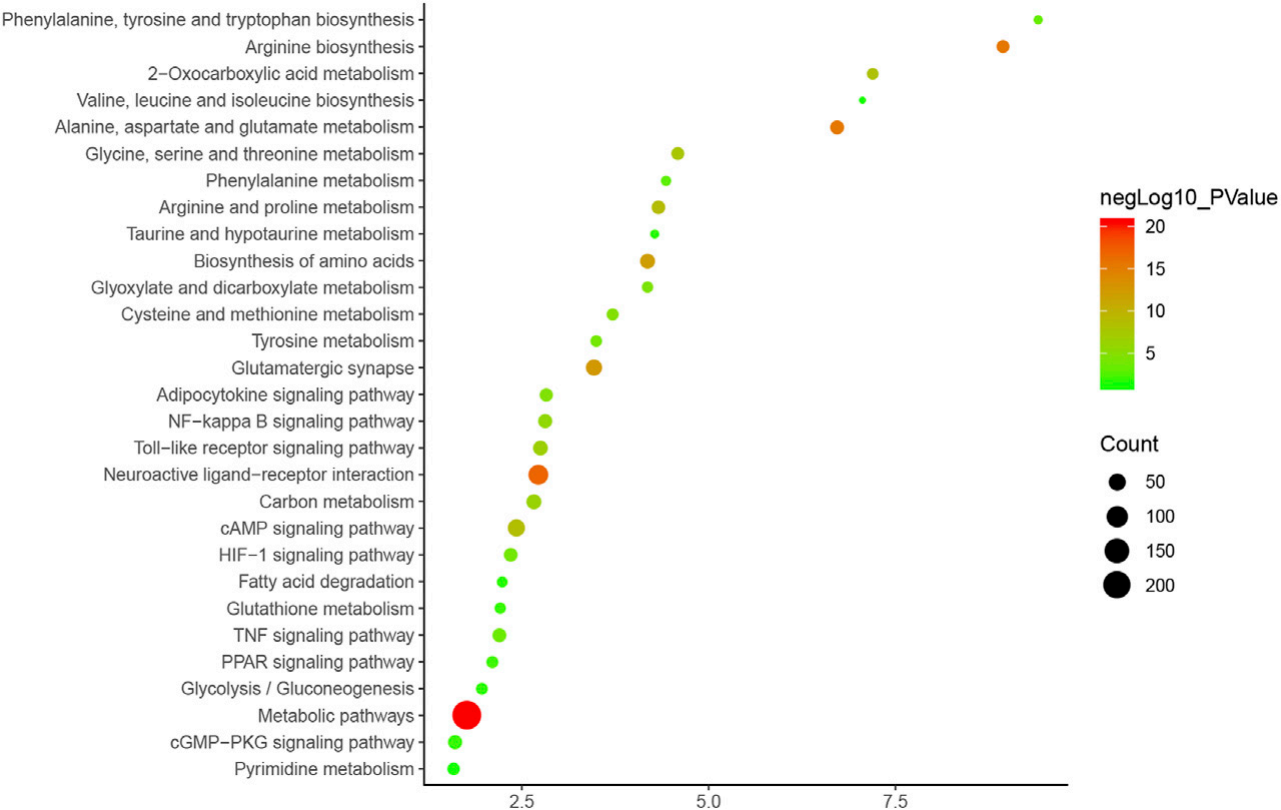
Bubble chart of signaling pathway (X-axis stand for fold enrichment).

The pathway enrichment analysis showed that ZBDHD can regulate a lot of orchitis -related signaling pathways, such as Metabolic pathways, Neuroactive ligand-receptor interaction, Arginine biosynthesis, Alanine, aspartate and glutamate metabolism, Glutamatergic synapse, Biosynthesis of amino acids, Arginine and proline metabolism, cAMP signaling pathway, 2-Oxocarboxylic acid metabolism, Glycine, serine and threonine metabolism (Figure 7 and Supplementary Table S4). The *p*-value, fold enrichment and count of each signaling pathways were shown in Figure 8.

### Evaluation of Establishment of UU Infection Rat Model

Seven days after the rat bladder was inoculated with UU, the testicular puncture fluid changed from yellow to red and the culture medium was transparent and clear after cultured in the UU liquid culture medium. After the solid medium was filtered through a microporous filter membrane, fried egg-like colonies were observed under a low power microscope, confirming that the rat testis tissue had UU growth, and the model was successful.

### UU Infection Rate in Rats

After treatment, rats in each group were sacrificed, and the epididymal tissue fluid was cut and cultured for UU. The results showed that UU was not cultured in the normal group, the positive rate of UU in the model group was 93.3% (14/15), the positive rate of UU in the ZBDHD group was 33.3% (5/15), and the positive rate of UU in the positive control group was 26.7% (4/15). Compared with the normal group, the positive rate of UU in the model group was higher, and the difference between the two groups was statistically significant (*p* < 0.01). Compared with the model group, the positive rates of UU in the ZBDHD group and the positive control group were reduced, and the difference was statistically significant (*p* < 0.05).

### Comparison of Rat Sperm Quality and Sperm Motility Parameters

**FIGURE 9:**
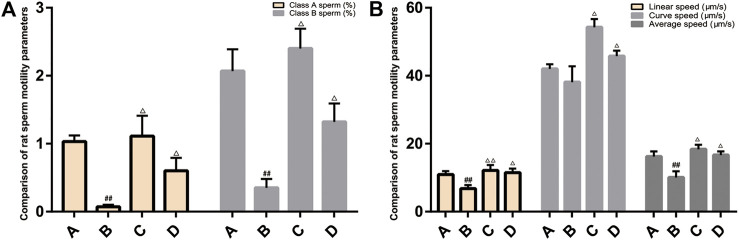
Comparison of rat sperm quality and sperm motility parameters. **(A)**: normal group; **(B)**: model group; **(C)**:ZBDHD group; D:positive control group. Compared with normal group ##*p* < 0.01; compared with model group △*p* < 0.05, △△*p* < 0.01).

Compared with the normal group, the rats in the model group had fast-moving sperm (A-grade sperm), slow-moving sperm (B-grade sperm), linear speed, and average speed decreased (*p* < 0.05, *p* < 0.01). After treatment, compared with the model group, the sperm motility parameters of each treatment group increased, and the difference was statistically significant (*p* < 0.05, *p* < 0.01) (Figure 9).

### Apoptosis of Rat Spermatogenic Cells

**FIGURE 10:**
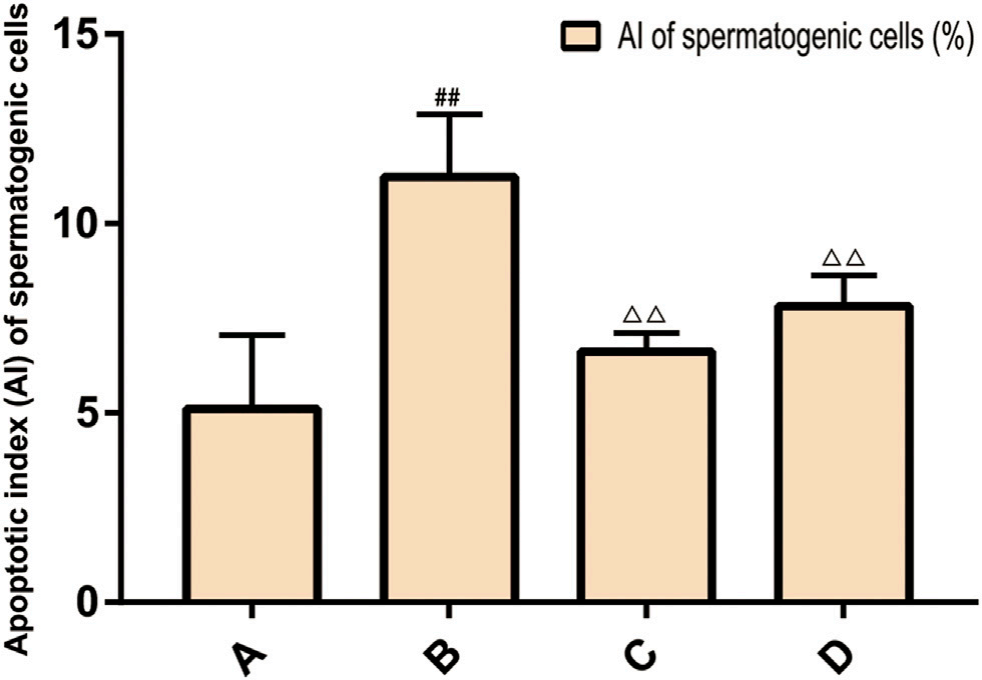
The AI of spermatogenic cells. **(A)**: normal group; **(B)**: model group; **(C)**: ZBDHD group; **(D)**:positive control group. Compared with normal group ##*p* < 0.01; compared with model group, △△*p* < 0.01).

**FIGURE 11:**
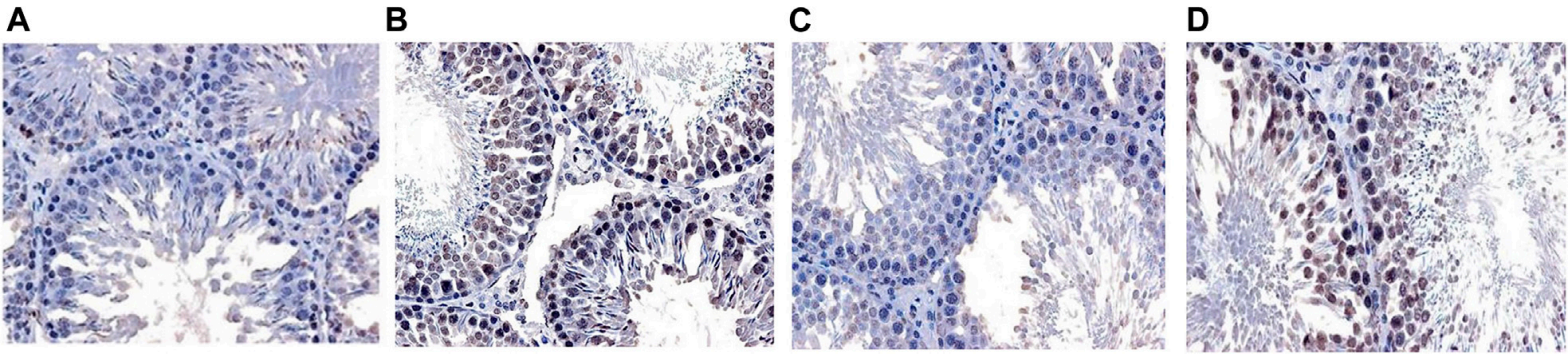
The Apoptosis of rat spermatogenic cells [TUNEL × 400. **(A)**: normal group; **(B)**: model group; **(C)**: ZBDHD group; **(D)**: positive control group].

The results of TUNEL method showed that there was spermatogenic cell apoptosis in the testicular tissues of each group, and the model group was the most serious. The AI of spermatogenic cells in the testis of the model group was significantly higher than that of the ZBDHD group and the positive control group (*p* < 0.01), while the AI of the positive control group was significantly higher than that of the ZBDHD group (*p* < 0.05) (Figures 10, 11).

### The Ultrastructure of Rat Spermatogenic Cell Mitochondria

**FIGURE 12:**
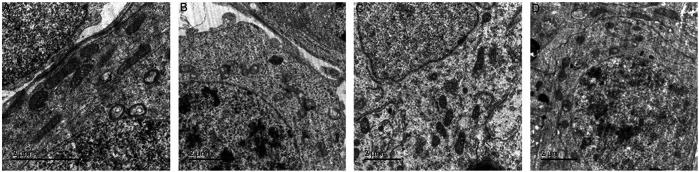
The ultrastructure of rat spermatogenic cell mitochondria [× 12,000 **(A)**: normal group; **(B)**: model group; **(C)**: ZBDHD group; **(D)**: positive control group].

In the normal group, the morphology and structure of spermatogenic cells at all levels were normal, and mitochondria could be observed in the cytoplasm with normal structure and abundant numbers and orderly arrangement. In the model group, spermatogenic cells at all levels showed obvious swelling and mitochondria with vacuolated ridges, and the membrane structure was unclear. In the positive control group, the arrangement, cell morphology and structure of spermatogenic cells were basically normal, and the size and number of mitochondria were basically normal; the mitochondria were slightly swollen but the mitochondrial cristae structure was still clear (Figure 12).

### Rat Spermatogenic Cell MMP

**FIGURE 13:**
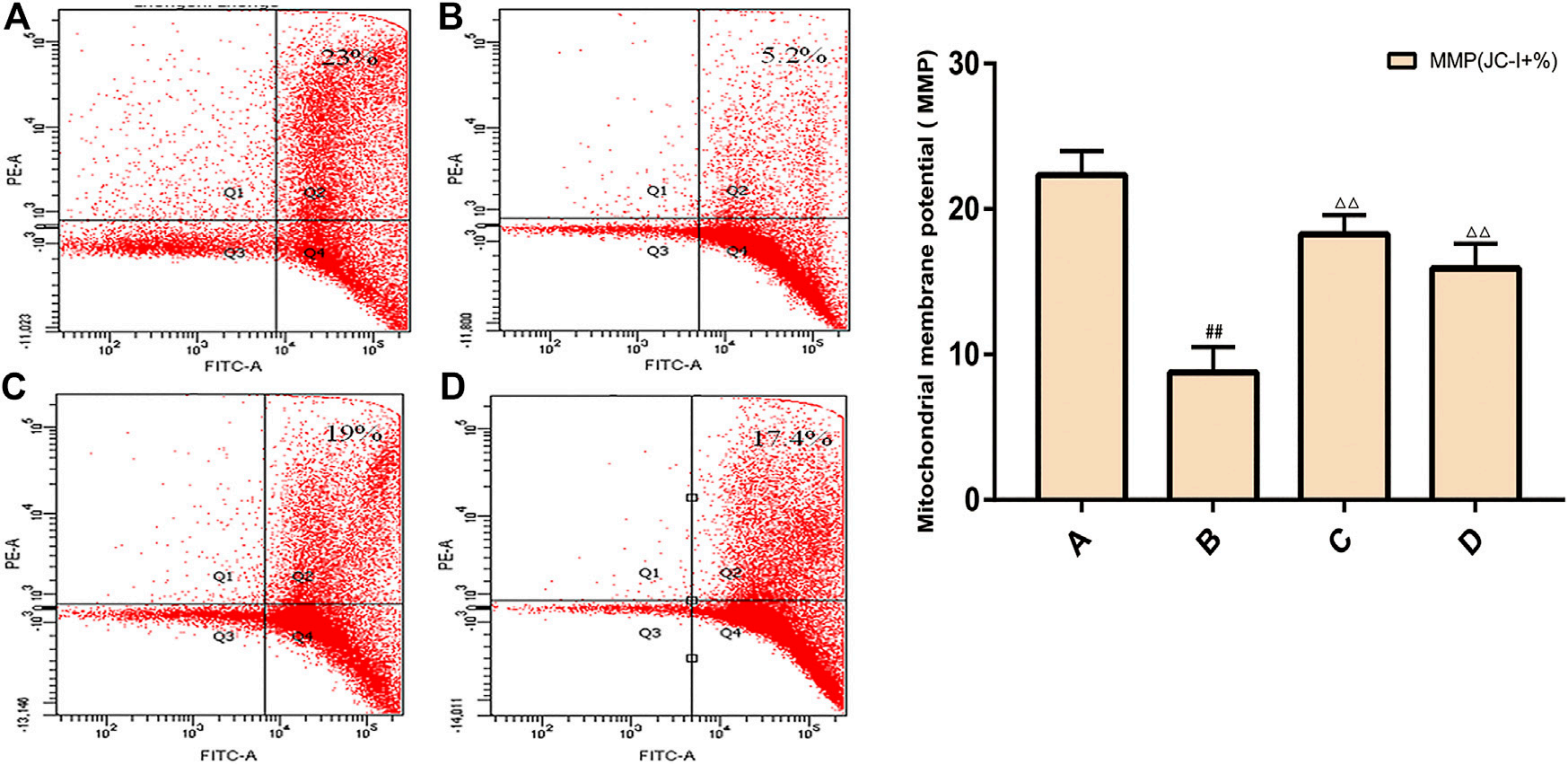
Rat spermatogenic cell MMP. **(A)**: normal group; **(B)**: model group; **(C)**: ZBDHD group; **(D)**: positive control group. Compared with normal group ##*p* < 0.01; compared with model group, △△*p* < 0.01.

The results of flow cytometry showed that the MMP level of spermatogenic cells in the model group was significantly lower than that of the control group (*p* < 0.01), and the MMP level of the ZBDHD group was significantly higher than that of the model group and the positive control group (*p* < 0.01) (Figure 13).

### Expression of MRPS22 mRNA and Protein in Rat Spermatogenic Cells

#### Expression of MRPS22 mRNA

**FIGURE 14:**
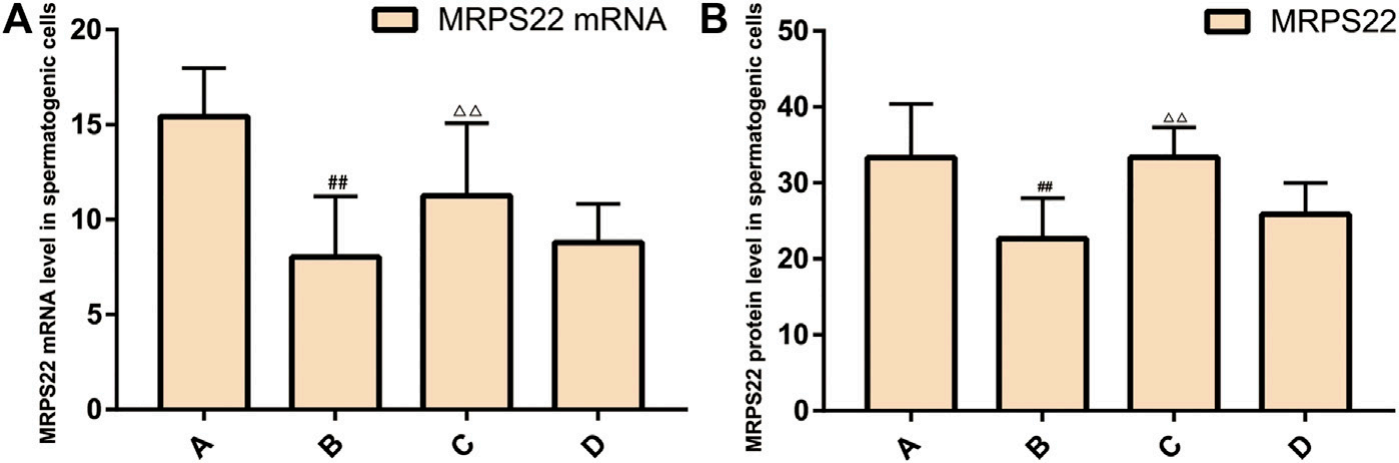
Expression of MRPS22 mRNA and proteins. **(A)**: MRPS22 mRNA; **(B)**: MRPS22 protein. A: normal group; B: model group; C: ZBDHD group; D: positive control group. Compared with normal group ##*p* < 0.01; compared with model group, △△*p* < 0.01.

The expression of MRPS22 mRNA was the lowest in the model group (*p* < 0.01), and the expression of MRPS22 mRNA in ZBDHD was significantly higher than that of the positive control group (*p* < 0.01) (Figure 14A).

#### Expression of MRPS22 Protein

**FIGURE 15:**
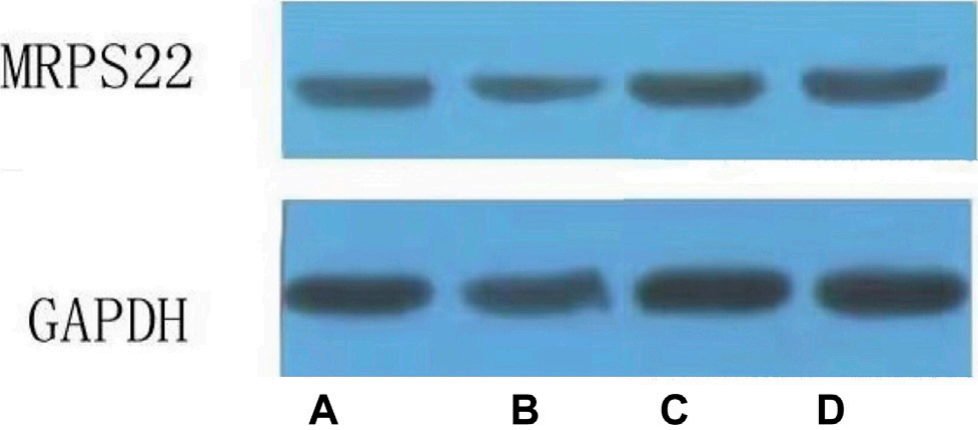
Expression of MRPS22 proteins. **(A)**: normal group; **(B)**: model group; **(C)**: ZBDHD group; **(D)**: positive control group.

The MRPS22 protein of the model group was significantly lower than that of the control group, ZBDHD group and the positive control group (*p* < 0.01). There was no significant difference between the MRPS22 protein of the ZBDHD group and the control group, but they were significantly higher than that of the positive control group (*p* < 0.01) (Figure 14B and Figure 15).

### Effect of ZBDHD on the Energy Metabolism Index of Rat Sperm

#### Methodological Quality Control

Under the aforementioned HPLC conditions, the precision, accuracy, stability, repeatability and recovery experiments were performed. The accuracy is controlled above 95%, the recovery rate is controlled above 95%, and the RSD is controlled within 4%, indicating that the instrument has good precision and reliable experimental methodology (Tables 2–4).

**TABLE 2:** Precision and accuracy (n = 6).

Standard	Concentration (mg/l)	Within-day			Inter-day		
		Measured value (mg/l)	RSD (%)	Accuracy (%)	Measured value (mg/l)	RSD (%)	Accuracy (%)
ATP	20	19.70	1.16	98.50	19.55	2.66	97.75
	50	49.25	1.36	98.50	49.20	1.86	98.40
	100	99.45	0.97	99.45	99.00	1.48	99.00
ADP	20	19.77	2.14	98.83	19.73	2.02	98.67
	50	49.52	1.29	99.03	49.37	2.06	98.73
	100	98.95	1.42	98.95	98.55	1.27	98.55
AMP	20	19.43	2.53	97.17	19.68	1.99	98.42
	50	49.22	2.03	98.43	48.78	2.06	97.57
	100	99.18	1.09	99.18	98.03	1.82	98.03

**TABLE 3:** Stability experiment and repeatability experiment RSD (%) (n = 5).

Standard	Stability	Repeatability
ATP	3 .60	2 .80
ADP	3 .49	1 .38
AMP	3 .36	2 .24

**TABLE 4:** Recovery rate experiment results (n = 2).

Analyte	Original amount (ug)	Adding amount (ug)	Measured amount (ug)	Recovery rate (%)	RSD (%)
ATP	71.42	20	89.89	98.33	2.38
		40	1 1 0.16	98.86	2.41
ADP	60.30	80	1 50.12	99.14	0.24
		20	80.99	100.85	1.96
		40	98.28	97.98	2.31
AMP	53.84	80	142.19	101.35	1.95
		20	73.96	100.16	1.54
		40	92.78	98.87	1.68
		80	1 33.06	99.41	3.09

#### Quantitative Analysis

Equal volumes of ATP, ADP, and AMP standard solutions are mixed and diluted into a series of working solutions for injection analysis. The standard curve is drawn (Supplementary Figure S1) with the concentration as the x-axis and the area under the peak area as the Y-axis:

1) ATP regression equation: y = 0.117x−0.077, correlation coefficient R2 = 0.9999 (Supplementary Figure S1A);
2) ADP regression equation: y = 0.104x+0.695, correlation coefficient R2 = 0.998 (Supplementary Figure S1B);
3) AMP regression equation: y = 0.128x−0.606, correlation coefficient R2 = 0.9998 (Supplementary Figure S1C).

Compared with the normal group, the sperm ATP and EC values of the model group decreased, and the AMP value increased (*p* < 0.01). Compared with the model group, the sperm ATP and EC values of the positive control group increased, while the AMP value decreased (*p* < 0.05, *p* < 0.01), while the AMP value of the ZBDHD group decreased (*p* < 0.05) (Supplementary Figure S2).

### Effect of ZBDHD on CytC in UU Infection Model Rat Sperm

Compared with the normal group, the CytC content of the model group and the positive control group was significantly increased (*p* < 0.01). Compared with the model group, the CytC content in the ZBDHD group decreased (*p* < 0.05). It shows that ZBDHD can inhibit sperm apoptosis and reduce the release of CytC from mitochondria in UU infection model rats (Supplementary Figure S3).

### Effect of ZBDHD on the Opening of Mitochondria mPTP in UU Infection Model Rat Sperm

Compared with the sham operation group, the absorbance of sperm mitochondria in the model group decreased (*p* < 0.01). Compared with the model group, the absorbance of sperm mitochondria of rats in each treatment group increased (*p* < 0.01) (Supplementary Figure S4).

### Effect of ZBDHD on the Activities of Na^+^-K^+^-ATPase and Ca^2+^-Mg^2+^-ATPase in UU Infection Model Rat Sperm

Compared with the sham operation group, the sperm Na^+^-K^+^-ATPase and Ca^2+^-Mg^2+^-ATPase activities of the model group were reduced (*p* < 0.01). Compared with the model group, the sperm Na^+^-K^+^-ATPase and Ca^2+^-Mg^2+^-ATPase activities of rats in each treatment group increased (*p* < 0.01) (Supplementary Figure S5).

## Discussion

The morphology, structure and function of sperm are important indicators to measure the ability of sperm to fertilize. Normal sperm structure and function are important conditions for fertilization (Manochantr et al., 2012). UU is adsorbed on the sperm surface, the lecithin lipid on the sperm membrane will be decomposed by the phospholipase A and C on the cell membrane, and the integrity of the sperm structure will be affected and it will fuse with the protoplasm. Toxic substances in the UU cytoplasm will enter the sperm cytoplasm, which will affect the development, maturation and normal physiological functions of the sperm, resulting in a decline in sperm motility and penetration ability (Xu et al., 1997; Wu et al., 2004). UU can survive and migrate in mammals, and can survive for a long period of time in testicular tissues, and can cause extensive lesions of seminiferous tubules. UU adheres to the spermatogenic cells, which can cause the latter to fall off the seminiferous tubules (Zhang et al., 2011; Qian et al., 2016). At the same time, UU can lead to the formation of multinucleated giant cells and sperm cell nucleus, seminal convoluted tubule atrophy, interstitial edema and exudation and other pathological changes, leading to oligospermia, azoospermia, and affecting sperm maturation. Meanwhile, UU infection can severely damage the morphology and function of sperm, and also make the acrosome membrane and the plasma membrane unable to fuse normally, the acrosome and the nuclear membrane are separated, the double-layer structure is partially interrupted, and the structure of part of the sperm head is damaged, which increases the rate of sperm deformity (Yang et al., 2018). UU infection has an impact on sperm density, sperm motility and forward motility, which can lead to a decrease in semen quality (Zhou et al., 2018).

Mitochondria regulate the energy production and apoptosis of eukaryotic cells, and provide energy for sperm movement. Normal mitochondrial structure is the basis for spermatogenic cells to exert their respiratory function to produce energy, which is directly related to the motility of sperm (du Plessis et al., 2015). UU infection can affect the structure and function of sperm mitochondria in many ways. UU enzymes and toxins can damage the sperm mitochondrial membrane, thereby affecting the production of sperm motility energy (Burrello et al., 2009). UU infection reduces the mitochondrial membrane potential (MMP) of sperm cells and hinders mitochondrial function (La Vignera et al., 2012). When the MMP of sperm cells decreases, the arrangement of mitochondria in the sperm tail will be disordered, the mitochondrial sheath will be missing, and the mitochondrial morphology and position will also be abnormal (La Vignera et al., 2012). UU infection will increase the content of reduced coenzyme oxidase that transfers single electrons in the reproductive system, and produce a series of reactive oxygen specie (ROS). ROS can promote the lipid peroxidation of unsaturated fatty acids on the inner and outer membranes of sperm mitochondria, resulting in sparse lipid arrangement of the membrane, reduced inner membrane ridges, and reduced ATP synthesis. MMP is negatively correlated with ROS content, so excessive production of reactive oxygen species in sperm is one of the important reasons for the decline of mitochondrial membrane potential (MMP). Sperm mitochondrial membrane may be damaged by reactive oxygen and insufficient energy supply and decreased vitality (La Vignera et al., 2012; Sellami et al., 2014). Mitochondrial energy synthesis will be affected by abnormal mitochondrial structure, so sperm motility and fertilization ability decrease (Agarwal and Said, 2005). MMP reflects the tricarboxylic acid cycle that generates energy in the mitochondria. Its decrease means that the mitochondrial energy supply is impaired, and it is a sensitive indicator for evaluating mitochondrial function. Studies have shown that there is a positive correlation between MMP and sperm viability, motility and sperm fertilization rate (Brahem et al., 2012). According to classical theory, the mitochondrial sheath in the middle of the sperm, synthesized by oxidative phosphorylation, is the source of energy required by the flagella of the sperm tail during sperm movement (Gong et al., 2017).

The permeability and fluidity of mitochondrial membrane are the guarantee for maintaining the structure and function of mitochondria. Abnormal mitochondrial permeability transition (MPT) can cause mitochondrial dysfunction. MPT is achieved through two mechanisms: the opening of the mPTP and the activation of Bax and Bak proteins to increase the permeability of the mitochondrial outer membrane (Baines et al., 2007). The abnormal opening of mPTP would cause the decrease of mitochondrial membrane potential, cell energy metabolism level and cell activity (Shen et al., 2014). mPTP is a transmembrane multi-protein complex pore, mainly composed of adenine nucleotide translocase (ANT) in the inner membrane, voltage-dependent anion channel (VDAC) in the outer membrane, cyclosporin A receptor D (cyclophilin-D, CyP-D) in the mitochondrial matrix (Lam et al., 2015). mPTP is normally closed to maintain the integrity of the mitochondrial structure, which is a necessary condition for mitochondria to undergo oxidative stress, ATP synthesis, and achieve their physiological functions (Lam et al., 2015). The synthesis of ATP and the electron transfer in the respiratory chain are carried out in the mitochondria. ATP and ADP are effectively exchanged and transported between the mitochondria and the cytoplasm, so that the cell respiration and the coupling of the respiratory chain are maintained normal (Halestrap and Brenner, 2003). The transport of ATP and ADP depends on the ANT/VDAC transporter complex formed by the adenine nucleotide carrier ANT and VDAC. The former is located in the inner mitochondrial membrane and the latter is located in the outer mitochondrial membrane (Halestrap and Brenner, 2003; Veenman et al., 2007). As a transmembrane protein with abundant content in the outer mitochondrial membrane, VDAC is involved in many physiological and pathological mechanisms, including energy metabolism and cell apoptosis (Vyssokikh and Brdiczka, 2003). In a certain pathological state, the continuous opening of the mitochondrial outer membrane VDAC reduces the mitochondrial inner membrane potential, that is, energy production decreases, respiratory function decreases, cytochrome C leaks, cell energy metabolism level decreases, and cell activity decreases. The excessive opening of mPTP leads to a decrease in the ratio of ATP/ADP, mitochondrial respiratory function is inhibited, the H^+^ gradient inside and outside the mitochondria disappears, the respiratory chain is uncoupled, and energy production is interrupted (Neginskaya et al., 2019). When the stimulation was mild, the number of irreversible openings of mPTP was small or it is temporarily opened and then closed again, and the intracellular ATP concentration did not decrease significantly, and apoptosis can only be induced by releasing CytC.

Our research shows that ZBDHD can improve sperm motility. After successful UU infection modeling, the proportion of sperm cells in the model group with decreased MMP was higher. After ZBDHD treatment, the proportion of sperm cells with decreased MMP in each group decreased. After successful UU infection modeling, the sperm ROS level of the model group was higher, which was significantly different from the normal group. After treatment with ZBDHD, the sperm ROS level of rats in each group decreased, which proved that ZBDHD can reduce the sperm ROS level of UU infection model rats and reduce the sperm ROS production of UU infection model rats. After successful modeling of UU infection, the content of CytC in sperm of the model group was higher, indicating that UU infection can increase sperm apoptosis and cause the release of CytC. After ZBDHD treatment, the content of CytC in sperm of rats decreased, suggesting that ZBDHD can inhibit sperm apoptosis and reduce the release of CytC from mitochondria in UU infection model rats.

In summary, the results of this study indicate that UU infection can lead to increased sperm MMP reduction ratio, ROS level and CytC content increase, and ZBDHD can reduce the production of sperm ROS, reduce the pathological damage of ROS to sperm membrane and function, and improve sperm MMP. This increases the energy synthesis of sperm mitochondria, reduces sperm apoptosis, and inhibits the release of CytC, so as to achieve the goal of anti-oxidation to increase sperm density, viability and vitality. This may be one of the mechanisms of ZBDHD in the treatment of male infertility caused by UU infection.

## Conclusion

This study discovered the multi-pathway mechanism of ZBDHD intervention in UU-induced orchitis through integrated pharmacological strategies, which provides a reference for further research on the mechanism of ZBDHD intervention in orchitis in the direction of mitochondrial energy metabolism. At the same time, it provides new research ideas for the research of classic famous TCM prescriptions.

## Data Availability

The original contributions presented in the study are included in the article/Supplementary Material, further inquiries can be directed to the corresponding authors.
