# Supplementary figures and images for: Exploring the Mechanism of Zhibai Dihuang Decoction in the Treatment of Ureaplasma Urealyticum-Induced Orchitis Based on Integrated Pharmacology
Source: Front Pharmacol. 2021 May 10;12:602543. doi: 10.3389/fphar.2021.602543 (PMC8141734; doi:10.3389/fphar.2021.602543)

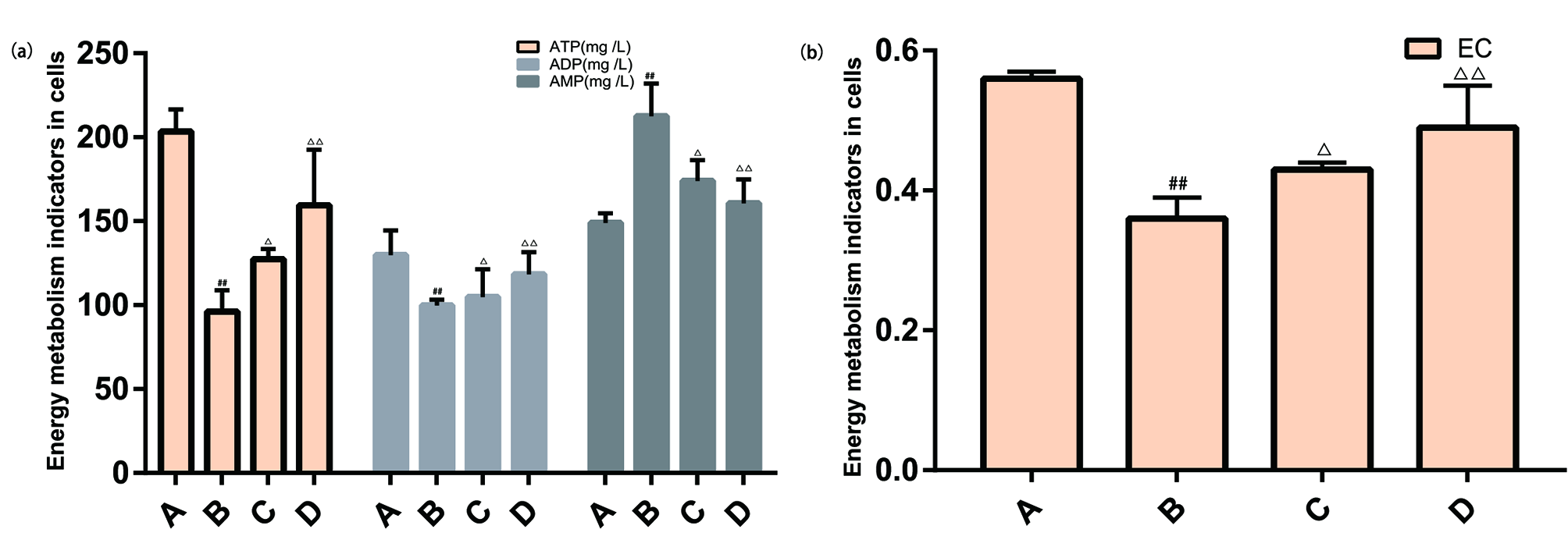

Supplement: Supplementary file 3 [file Image3.TIF]

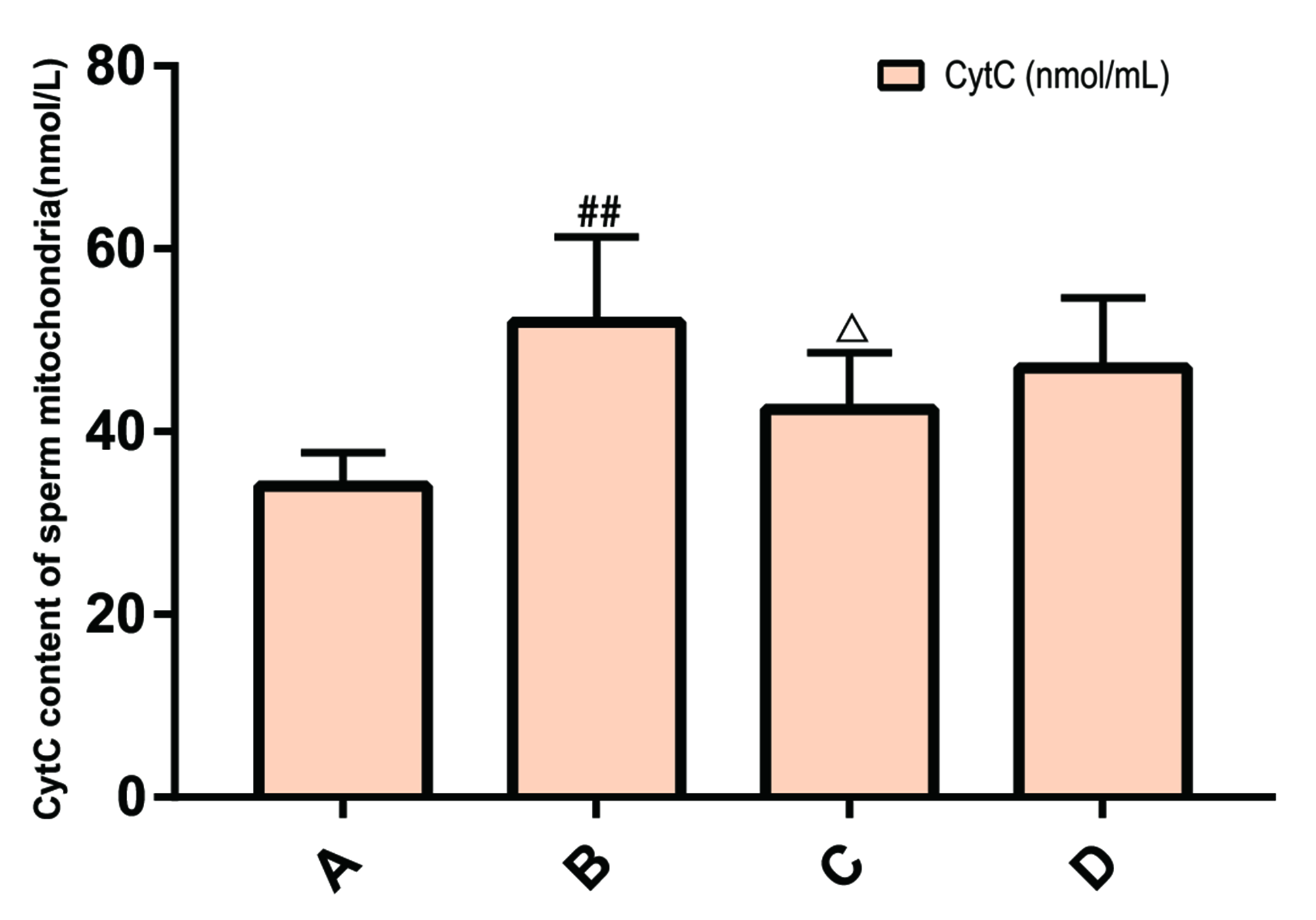

Supplement: Supplementary file 4 [file Image4.TIF]

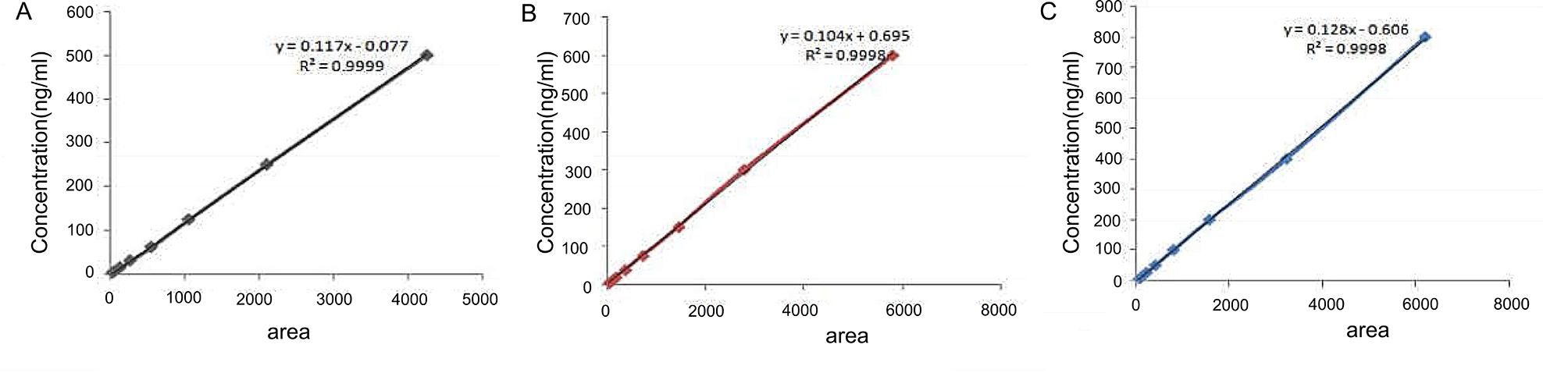

Supplement: Supplementary file 5 [file Image2.TIF]

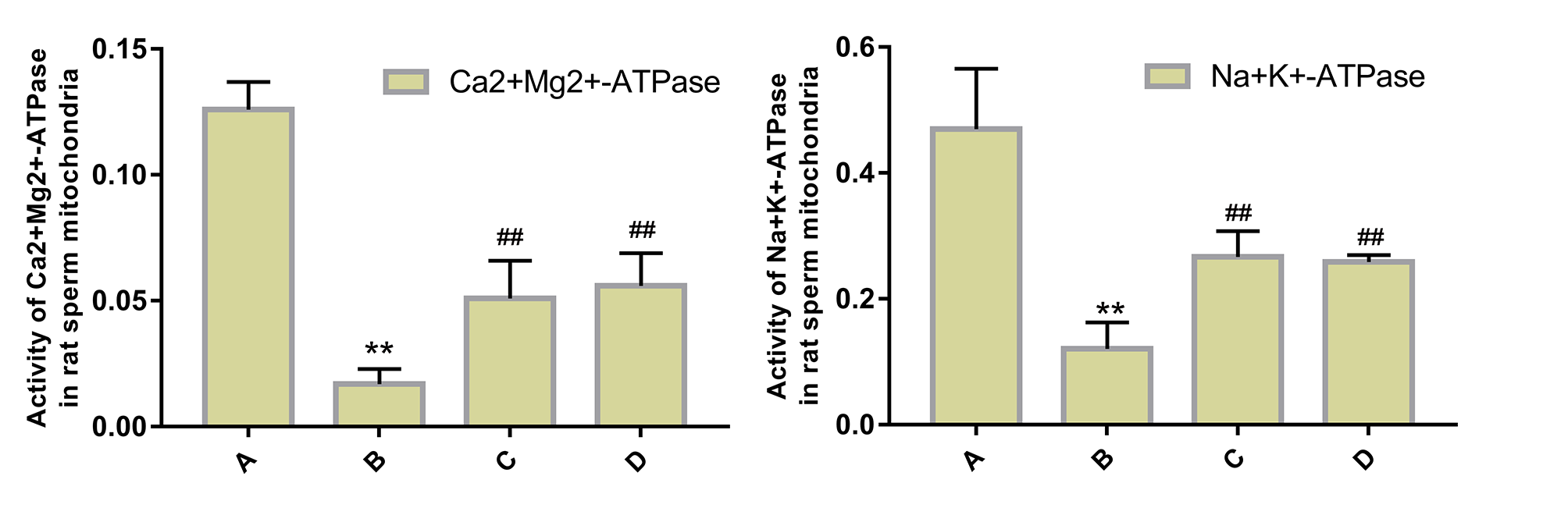

Supplement: Supplementary file 6 [file Image1.TIF]

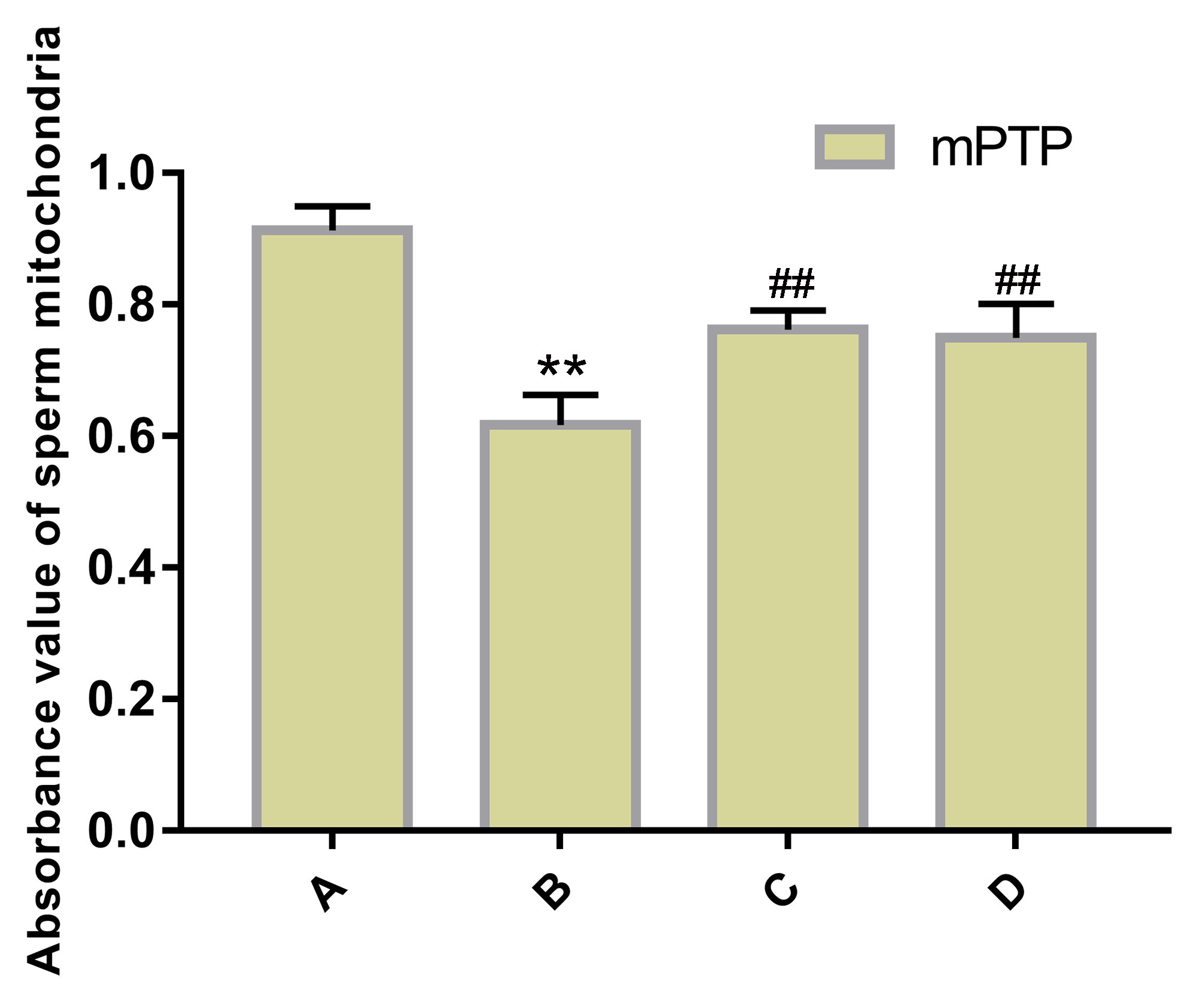

Supplement: Supplementary file 9 [file Image5.TIF]
